# Supplementary material for: Resolving the full spectrum of human genome variation using Linked-Reads
Source: Genome Res. 2019 Apr;29(4):635–45. doi: 10.1101/gr.234443.118 (PMC6442396; doi:10.1101/gr.234443.118)
Supplement: Supplemental Material [file supp_gr.234443.118_Supplemental_longranger-2.2.zip › longranger-2.2.tar.bz2!/lr2.2-public/lib/stan/cmdstan/stan/doc/api/html/classstan_1_1io_1_1stan__csv__reader.html]

Stan: stan::io::stan\_csv\_reader Class Reference


|  |  |
| --- | --- |
|  | Stan  2.5.0  probability, sampling & optimization |


- Main Page
- Related Pages
- Namespaces
- Classes
- Files

- Class List
- Class Index
- Class Hierarchy
- Class Members

All Classes Namespaces Files Functions Variables Typedefs Enumerations Enumerator Friends Macros Pages

- stan
- io
- stan\_csv\_reader

Public Member Functions |
Static Public Member Functions |
List of all members

stan::io::stan\_csv\_reader Class Reference

Reads from a Stan output csv file.
More...

`#include <stan_csv_reader.hpp>`

|  |  |
| --- | --- |
| Public Member Functions | |
|  | stan\_csv\_reader () |
|  | |
|  | ~stan\_csv\_reader () |
|  | |

|  |  |
| --- | --- |
| Static Public Member Functions | |
| static bool | read\_metadata (std::istream &in, stan\_csv\_metadata &metadata) |
|  | |
| static bool | read\_header (std::istream &in, Eigen::Matrix< std::string, Eigen::Dynamic, 1 > &header) |
|  | |
| static bool | read\_adaptation (std::istream &in, stan\_csv\_adaptation &adaptation) |
|  | |
| static bool | read\_samples (std::istream &in, Eigen::MatrixXd &samples, stan\_csv\_timing &timing) |
|  | |
| static stan\_csv | parse (std::istream &in) |
|  | Parses the file. More... |
|  | |

## Detailed Description

Reads from a Stan output csv file.

Definition at line 71 of file stan\_csv\_reader.hpp.

## Constructor & Destructor Documentation

|  |  |  |  |  |
| --- | --- | --- | --- | --- |
| stan::io::stan\_csv\_reader::stan\_csv\_reader | ( |  | ) |  |

inline

Definition at line 75 of file stan\_csv\_reader.hpp.

|  |  |  |  |  |
| --- | --- | --- | --- | --- |
| stan::io::stan\_csv\_reader::~stan\_csv\_reader | ( |  | ) |  |

inline

Definition at line 76 of file stan\_csv\_reader.hpp.

## Member Function Documentation

|  |  |  |  |  |  |
| --- | --- | --- | --- | --- | --- |
| static stan\_csv stan::io::stan\_csv\_reader::parse | ( | std::istream & | *in* | ) |  |

inlinestatic

Parses the file.

Definition at line 319 of file stan\_csv\_reader.hpp.

|  |  |  |  |
| --- | --- | --- | --- |
| static bool stan::io::stan\_csv\_reader::read\_adaptation | ( | std::istream & | *in*, |
|  |  | stan\_csv\_adaptation & | *adaptation* |
|  | ) |  |  |

inlinestatic

Definition at line 183 of file stan\_csv\_reader.hpp.

|  |  |  |  |
| --- | --- | --- | --- |
| static bool stan::io::stan\_csv\_reader::read\_header | ( | std::istream & | *in*, |
|  |  | Eigen::Matrix< std::string, Eigen::Dynamic, 1 > & | *header* |
|  | ) |  |  |

inlinestatic

Definition at line 155 of file stan\_csv\_reader.hpp.

|  |  |  |  |
| --- | --- | --- | --- |
| static bool stan::io::stan\_csv\_reader::read\_metadata | ( | std::istream & | *in*, |
|  |  | stan\_csv\_metadata & | *metadata* |
|  | ) |  |  |

inlinestatic

Definition at line 78 of file stan\_csv\_reader.hpp.

|  |  |  |  |
| --- | --- | --- | --- |
| static bool stan::io::stan\_csv\_reader::read\_samples | ( | std::istream & | *in*, |
|  |  | Eigen::MatrixXd & | *samples*, |
|  |  | stan\_csv\_timing & | *timing* |
|  | ) |  |  |

inlinestatic

Definition at line 242 of file stan\_csv\_reader.hpp.

---

The documentation for this class was generated from the following file:

- src/stan/io/stan\_csv\_reader.hpp


---

[ Stan Home Page ]

*© 2011–2014,
Stan Development Team.*
